# Knowledge, attitudes, and practices towards mosquito control and used vehicle tire dumping by median household income, in metropolitan New Orleans, Louisiana

**DOI:** 10.7717/peerj.14188

**Published:** 2022-12-02

**Authors:** Imelda K. Moise, Ashley Archer, Claudia Riegel

**Affiliations:** 1 Department of Geography & Sustainable Development, College of Arts & Sciences, University of Miami, Coral Gables, FL, United States of America; 2 New Orleans Mosquito, Termite and Rodent Control Board, City of New Orleans, New Orleans, LA, United States of America

**Keywords:** Mosquito, Waster tires, Littering, Recycling, Culex, Breeding, USA

## Abstract

**Background:**

Discarded vehicle tires are an important artificial habitat for the larvae of many container-breeding mosquito species worldwide, including in the United States. Unmanaged discarded vehicle tires create health, environmental and social costs, and with budget and staffing constraints, effective management of discarded used vehicle tires a mosquito larval habitat depends in part on the knowledge, attitude, and practices (KAP) of community residents.

**Objectives:**

This study aims to examine the knowledge, attitude and practices of New Orleans, Louisiana residents toward illegally discarded vehicle tires, and larval mosquito control.

**Methods:**

A descriptive cross-sectional design study was used where 422 households were selected using a two-stage cluster random sampling procedure in New Orleans, Louisiana. Heads of households or a person aged 18 years or older self-administered the survey. The questionnaire comprised five parts: screening, tire sightings, preferred communication method, knowledge, attitude and precautionary measures against mosquito control, disease risk and illegal discarding. We then statistically compared above and below median income household responses to identify likely causes of detected differences. The data were analyzed using ordinal regression models via IBM SPSS statistics V.26.0. Statistical significance was set at *p* < 0.05.

**Results:**

Out of 290 responding households, 95.5% strongly agree or agree that mosquitoes can spread serious diseases like West Nile, Zika or Dengue. Only 2.3% of the sample had high knowledge of illegally discarded tires dumping and mosquito larval control. Those employed were 1.0 times more likely to possess good knowledge than the unemployed (*p* < 0.001). Despite low knowledge levels regarding mosquito breeding and polluted water in discarded tires, 29.9% of respondents had positive attitude and 20.5% reported sufficient practices. Among the socio-demographic variables, only home ownership and being employed were predictors of knowledge and attitude towards mosquito breeding in illegally discarded tires (*p* < 0.05).

**Conclusions:**

Despite the observed increasing number of illegally discarded vehicle tires in New Orleans, the knowledge of people about illegal tire dumping and their associated risk factors as suitable larval habitants was low. Therefore, there is a need for developing community-based and place-based tailored sensitization campaigns to prevent illegal used tire dumping, and larval control.

## Introduction

Discarded vehicle tires are a major problem in both urban and rural areas in continental US (Kling, Juliano & Yee, 2007; Yee, 2008). A key issue is the disposal of waste or used vehicle tires generated at one location and disposed of at another location without legal permission. Extensive research has shown that such discarding often occurs near human dwellings and activity (Aziz et al., 2012; Baumgartner, 1988; Beier et al., 1983). The unmanaged discarded waste vehicle tires pose risks to population and environmental health and can create social costs (Chen et al., 2009). For example, it has previously been observed that accumulated waste vehicle tires when left exposed to the elements continually deteriorate environmental quality (Remoundou & Koundouri, 2009). In addition, the durability of discarded vehicle tires makes tires a suitable larval habitat for important disease-vectoring mosquitoes (Beier et al., 1983; Yee, Kneitel & Juliano, 2010). Mosquitoes reported as favoring breeding in accumulated tires are recognized worldwide as vectors of arboviruses (Yee, Kneitel & Juliano, 2010; Moise et al., 2021; Fernandes et al., 2018), and include those in the genera *Aedes* (*e.g.*, *Aedes albopictus* (Skuse, 1895), *Japonicus* (Theobald, 1901) and *Culex* (*e.g.*, *Culex restuans* (Theobald, 1901), *Culex pipiens* (Linnaeus, 1762), *Culex territans* (Walker, 1856), *Culex salinarius* (Coquillett, 1902) and *Culex quinquefasciatus* (Say, 1823)) (Yee, Kneitel & Juliano, 2010; Haramis, 1984; Aliabadi & Juliano, 2002; Costanzo, Mormann & Juliano, 2005; González et al., 2020; Peyton et al., 1999; Hawley et al., 1987).

In New Orleans, Louisiana, discarded vehicle tires are emerging as an important artificial source of species that normally reproduce in containers such as *Aedes albopictus*. Our previous study on this topic conducted in 2020 identified 11 species in five genera from discarded tires sampled from November 2016 to January 2018. The most abundant and widely distributed species were *Ae. albopictus* (Container Index (CI) of 44.19%) and followed by *Cx. quinquefasciatus* (CI, 22.18%) (Sallam et al., 2017). The problem of illegally discarded vehicle tires in vacant properties in the city (Lewis et al., 2017), and larval occupancy escalated in response to lingering Hurricane debris (Congressional Research Service, 2008; Weave, 2006), a lack of regulation for waste tire businesses (new or used tires) (State of Louisiana, 2011; City of New Orleans, 2019), and limited options for eventual disposal (only one Department of Environmental Quality approved tire processor existed at the time of writing this paper) (Weave, 2006). Meanwhile, collection of illegally discarded vehicle tires from public right of ways has doubled from 21,084 in 2010 to 47,927 in 2018 (unpublished City of New Orleans Department of Sanitation records). Additionally, although the City of New Orleans Mosquito, Termite and Rodent Control Board larvicide mosquito breeding in discarded vehicle tires as a part of routine mosquito control operations, beginning in 2018, larvicide treatments has been rare. This is mainly due to the increasing number of illegally discarded vehicle tires, inadequate staffing levels and the burden posed on the Board’s operational budget.

With budget and staffing constraints, effective management of illegally discarded tires, and larval control depends in part on the knowledge, attitude, and practices (KAP) of community residents (Davis, Cook & Cohen, 2005; Farquhar, Michael & Wiggins, 2005; Bodner et al., 2016). However, while KAP surveys relating to mosquito control outcomes, environmental health, human well-being has been carried out in Africa (Kala Chouakeu et al., 2021; Manga et al., 2021), Asia (Regmi, Kunwar & Ortega, 2016; Phuyal et al., 2022; Selvarajoo et al., 2020; Corrin et al., 2017) and the Americas (Elson et al., 2020) where mosquito disease risk is more pronounced, few have been carried out in the US (Dowling et al., 2013; Cherry et al., 2016). Most of the conducted studies have used qualitative study designs with a mix of descriptive, cross sectional and exploratory research design tools and have focused on assessing KAP’s relationship to socioeconomic status and mosquitoes (Dowling et al., 2013; Walker et al., 2018) rather than on source reduction. For instance, one study conducted in upstate New York demonstrated that residents’ perceptions of WNV as a serious disease had a significant role in shaping their prevention practices (*e.g.*, fewer positive containers in residential yards) (Tuiten et al., 2009). Similar findings were noted by a study conducted in Arizona that assessed public perceptions of non-pharmaceutical interventions for influenza and mosquito-borne illnesses which noted low knowledge among residents regarding mosquito-borne diseases (Pogreba-Brown et al., 2019). In New Orleans, and because most of the illegal tire discarding is a result of opportunist dumping in fields and vacant properties mostly for convenience and/or to avoid paying dumping fees (WGNO, 2020), it is reasonable to expect varying knowledge and practices among the surveyed households. Further, KAP survey evidence in Washington, DC linked adequate knowledge of mosquito development to source reduction and lower numbers of *Ae. albopictus*-positive containers (Dowling et al., 2013).

As the numbers of illegally discarded tires increases each year in New Orleans, gaining an in-depth knowledge of the ecological, biological, and social (eco-bio-social) factors that contribute to the relative abundance of mosquito populations in the city is key to developing effective strategies on how to advance diverse mosquito control efforts. These lessons could help to better guide the development of community-based and place-based tailored outreach strategies to monitor and mitigate illegal tire discarding and mosquito control. Such findings can also be used in other urban or peri-urban environments facing similar challenges. Here, we examined the knowledge and attitude of New Orleans, Louisiana residents (at household level) toward mosquito control and illegal vehicle tire discarding including associated disease risks. We hypothesize that educated households and those with higher incomes will be more willing to report illegal tire discarding, have better practices that prevent mosquitoes from breeding and biting as well as to be more knowledgeable of the disease risks posed by the accumulation of waste tires.

### Ethics

The project received ethical exemption from the University of Miami Institutional Review Board (IRB ID: 20161212).

## Methods

### Study design

A descriptive cross-sectional design study of New Orleans, Louisiana households residing in affluent neighborhoods (*n* = 10) and low-income neighborhoods (*n* = 71). The city of New Orleans has 73 distinct neighborhoods grouped into 13 planning districts, and approximately 61 Zip Codes (New Orleans City Planning Commission, 2016), and illegal used vehicle tire discarding continue to be a problem (WDSU, 2021). Data were collected from November 2018 through October 2019. Households were recruited via internet, survey, and during neighborhood engagement meetings.

### Study settings

Orleans Parish and the City of New Orleans are coterminous (Moise, Riegel & Muturi, 2018), and is located at 105 miles upriver from the Gulf of Mexico, and in the Mississippi River Delta, south of Lake Pontchartrain, on the banks of the Mississippi River. It is located at 90°4′14″W and latitude 29°57′53″N, with an elevation range of −6.5 to 20 ft. Mosquito season usually begins in the spring (March) and slows down in the fall (November to December) depending on temperature.

### Study sample

First, to select study households, home addresses of New Orleans households were purchased from Infogroup (now Data Axle, Papillion, NE, USA). Second, a two-stage cluster random sampling procedure was used to calculate a sampling interval (SI) by dividing the total population in the study area (33,870) into 24 clusters selected from 61 New Orleans Zip Codes using a probability proportional to population size. Cumulative Zip Code population sizes were calculated by size of each Zip Code’s population, with a range developed for each Zip Code. To determine the clusters, first, we randomly selected a number between 1 and a calculated sampling interval of 3,354.3. The Zip Code with the selected number (*e.g.*, 12) was used as the first sampling cluster. The second cluster was determined by adding the sampling interval to this number—a process that continued until 24 clusters were identified. In stage 2 and to avoid bias, the office secretary assigned home addresses on small pieces of papers from which the sample of households per Zip Code were selected per cluster/Zip Code with envelops assigned with the unique identification numbers. Third, Cochran’s formula (Smart Methodology, 2012) was used to estimate a sample size with a power of 80% with a 5% margin of error and 95% confidence interval. Because the prevalence of KAP in the New Orleans communities is new, a sample size was estimated by assuming that 50% of the population has baseline knowledge about illegal tire discarding and the role that tires serve as an important habitat for mosquito larvae as was calculated in a related study (Selvarajoo et al., 2020). A minimum of 384 completed surveys were required for this study: with an additional 10% to capture non-response for a final sample of 422 households.

### Study participants

All households aged at least 18 years or older (mostly heads of households consenting to take the survey) and providing informed consent completed the survey. In cases where the head of household was absent, a person aged 18 years or older completed the survey. We initially pilot tested the questionnaire with 10 participants at neighborhood engagement meetings. The research was conducted in line with the guidelines provided towards human subject research compliance with written informed consent forms.

### Survey instrument

To collect data, we utilized a self-administered, 62 itemed questionnaire comprising of five sections developed using a combination of questions from various KAP surveys, and the first author’s surveys (Moise, Sheskin & Fuller, submitted). In addition to the three screening questions (residency status, age, length of residence), the questionnaire included 12 questions about tire sightings, tires in own yard, willingness to report if an illegal dumping is observed and to participant in a cleanup effort (very unlikely, unlikely, somewhat likely, likely). Ten questions addressed preferred communication method for receiving information about public health and tire dumping, seven questions addressed perceived effective education strategies and 12 questions addressed knowledge towards mosquitoes and disease risk (*e.g.*, diseases transmitted by mosquitoes, mode of transmission, time of day when mosquitoes are more likely to bite, city tire pick-up every second of the week (yes, no)), frequency of being bite around the home (very frequently, frequently, occasionally, and seldom or never), and mosquito-breeding sites.

To measure attitudes related to mosquito and illegal tire disposal (seven questions) comprised the perceptions towards control measures (reducing mosquito populations could avoid mosquito borne disease, mosquito borne diseases a serious health problem, spraying is the best method to reduce mosquito populations, the City of New Orleans is responsible for reducing the number of mosquito larvae, it is my individual responsibility to prevent the spread of mosquito, I am willing to report an illegal dumping if i see one and I am willing to participate in tire clean-up efforts). Strongly agree to agree responses were combined and assigned a score of 1 where responses yielding strongly disagree or disagree were assigned a score of 0.

Preventative behavior practices were measured using nine items covering two categories: (1) source reduction measures (five questions *e.g.*, applying bleach and/or detergents to water basins and water containers, eliminate stagnant water, fumigate, apply larvicide, use of fishes on water containers, and cover or empty water containers), and (2) preventing mosquito bites (four questions *e.g.*, use of insecticide, repellents, screens in window and doors, fans, and bed nets). These questions also yielded yes or no responses. The questionnaire was the tested for its reliability and validity with participants at neighborhood engagement meetings, with internal consistency evaluated using Cronbach’s alpha (*α* = 0.78) (Smart Methodology, 2012), and piloted with 10 participants.

Socio-demographic and housing variables of interest included age, sex, race/ethnicity, highest level of education, home five-digit Zip Code, marital status, current employment status, household income, type of current housing (owner occupied, rental, other), knowledge (*n* = 3 questions), attitudes (*n* = 4 questions), and practices (*n* = 2 questions). Respondent sex was classified and coded as male, 0, female, 1 and other, 3. Because only respondents aged 18 years or older completed the survey, age which was collected as a continuous variable was categorized into four categories 18–24: 0, 25–34: 1, 35–44: 2, 45+, 3 and missing, 999. Because the survey was purposefully designed to distinguish those who achieved higher education from those who did not, education was dichotomized into four categories less than a high school diploma: 0, some college: 1, bachelor’s degree: 2, and graduate degree: 3. Marital status was classified and coded as single (never married): 0, married or in a domestic partnership: 1, widowed, divorced, or separated: 2. Employment status was classified and coded as employed full-time: 0, employed, part-time: 1, unemployed: 2, retired or disabled: 3. Homeownership was categorized and coded as owner occupied: 0, rental: 1, other: 2, and property type was classified and coded as single-family home: 0, duplex: 1, apartment: 2, condominium: 3 and other property types: 4.

We delineated household income based on the reported participant household annual income and residence Zip code. In New Orleans, the lowest zip code household income value is $7,448, and the highest value is $73,042. In the sample, half of the values have household incomes between $19,000 and $30,000. The middle (median) household income value was $45,615. Based on this, KAP study households (respondents) were classified into two distinct median household income ranges; low (respondents with household incomes above the New Orleans middle (median)) household income of $45,615, and high-income (respondents whose median household income was above the median).

### Statistical analysis

Descriptive statistics of socio-demographic and housing factors of respondents were calculated and presented as frequency (percentage (%) for categorical variables) and as mean (M) ± standard deviation (SD, for continuous variables). Univariate analysis was realized by creating contingency tables and then performing Chi-square test. To compare the KAP levels (good, moderate, or poor) and the variables of interest by median household income, the effect size was estimated using Cramer’s V (for R*C tables) or Phi (for 2 × 2 tables).

Regarding knowledge, attitudes, and practices (KAP) outcome variables (KAP scores), first we assigned each respondent’s correct answer to KAP domain questions a score of ‘1’ and a ‘0’ to each wrong response. Second, we summed each respondent’s correct KAP responses and calculated their KAP scores. A cut-off point of 80.0–100.0%, 60.0–79.0%, and ≤59.0% was used to categorize each respondent’s overall KAP score. Because the overall KAP scores were highly skewed and discontinuous, we transformed the KAP scores into ordinal variables (poor, moderate, good), with a good score ranging between 8 and 10 points, a moderate score ranged from 6 and 7.9 points and a poor score was any respondents scoring less than <6 points as used in previous related KAP studies (Flatie & Munshea, 2021; Moise et al., 2018). For instance, with a total of 12 questions in the knowledge domain, a respondent scoring 10 or more scores was classified as having good level of knowledge, and this was true for attitude and preventative measures (negative, positive, or moderate attitude).

Because our dependent variables (knowledge and levels) were ordinal, we applied cumulative link models, also known as ordinal regression models. To account for the pairwise aspect of the data and possibility of correlation effect on the dependent variable levels, we used a PLUM (Polytomous Universal Model) ordinary regression model. The Zip Code of residence was set as random intercept in each model to denote contextual effects and a possible group effect. Covariates on interest include sex, education status, marital status, employment status, median household income, and home ownership. The likelihood ratio statistic, with chi-square statistic were used to select the final model using an automated step function. The data were analyzed using IBM SPSS statistics V.26.0 (IBM, Armonk, NY, USA). The level of statistical significance was set at *p* < 0.05.

## Results

### Characteristics of sample

Of the 422 solicited households, 290 households completed the surveys (76.3% participation rate). The median age of respondents was 49.5 years (SD = 14.79). More than half of respondents were females (57.2%), and 49.0% were Black or White (29.0%). A bachelor’s degree was the highest level of education attained by 33.4% respondents, 30.3% had a graduate or professional degree, and 18.3% had attained an associate degree or a high school diploma (5.9%). Of the respondents nearly half (45.9%) were married, and 29.3% were single or never married, owned homes (63.5%), and 45.5% reported living in single-family homes. Among the respondents, over half (51.0%) were employed fulltime, and median household income for more than one-third (36.9%) of respondents was over $75,000 (36.9%), followed by those earning between $20,000 and $49,999 (30.3%). Additionally, respondents indicated that it was very frequent (25.9%) or frequent (16.9%) as well as occasionally (40.3%) to experience mosquito bites (Table 1).

**Table 1:** Socio-demographic factors of participating households (*n* = 290).

**Variables**	**High-income residents** ***n* (%)**	**Low-income residents** ***n* (%)**	**Missing** ***n* (%)**	**Total** ***N* (%)**	***p*-value**
**Sex**					0.001
Male	57 (33.3)	21 (23.9)	0 (0.0)	78 (26.9)	
Female	103 (60.2)	62 (70.5)	1(3.2)	166 (57.2)	
Other (did not specify)					
**Age group (years)**					0.001
18–24	9 (5.3)	4 (4.5)	0 (0.0)	13 (4.5)	
25–34	20 (11.7)	14 (15.9)	1 (3.2)	35 (12.1)	
35–44	38 (22.2)	14 (15.9)	0 (0.0)	52 (17.9)	
45–54	42 (24.6)	12 (13.6)	0 (0.0)	54 (18.6)	
Over 55	58 (33.9)	41 (46.6)	0 (0.0)	99 (34.1)	
**Race/Ethnicity**					
Asian	4 (2.3)	2 (2.3)	0 (0.0)	6 (2.1)	0.001
Black	84 (48.0)	55 (62.5)	5 (16.1)	142 (49.0)	
Hispanic/Latino	3 (1.8)	1 (1.1)	0 (0.0)	4 (1.4)	
White	65 (38.0)	18 (20.5)	1 (1.1)	84 (29.0)	
Two or more races	10 (5.8)	9 (10.2)	0 (0.0)	19 (6.6)	
American Indian or Alaska Native	1 (0.6)	1 (1.1)	0 (0.0)	2 (0.7)	
**Education status**					0.001
Less than a high school diploma	4 (2.3)	13 (14.8)	0 (0.0)	17 (5.9)	
Some college	18 (10.5)	35 (39.8)	0 (0.0)	53 (18.3)	
Bachelor’s degree (e.g., BS., BA)	75 (43.9)	22 (25.0)	0 (0.0)	97 (33.4)	
Graduate degree (e.g., MA. MSc, PhD, JD, MD)	73 (42.7)	15 (17.0)	0 (0.0)	88 (30.3)	
**Marital status**					0.001
Singe (never married)	42 (24.6)	42 (47.7)	1 (3.2)	85 (29.3)	
Married or in a domestic partnership	105 (61.4)	28 (31.8)	0 (0.0)0	133 (45.9)	
Widowed, divorced, or separated	22 (12.9)	17 (19.3)	1 (1.1)	40 (13.8)	
**Home ownership**					
Owner occupied	131 (76.6)	51 (58.0)	2 (6.5)	184 (63.4)	
Rental	33 (19.3)	29 (33.0)	4 (12.9)	66 (22.8)	
Other (e.g., not specified)	4 (2.3)	4 (4.5)	0 (0.0)	8 (2.8)	
**Property type**					0.001
Single family home	76 (44.4)	54 (61.4)	2 (6.5)	132 (45.5)	
Duplex	15 (8.8)	5 (5.7)	0 (0.0)	20 (6.9)	
Apartment	6 (3.5)	5 (5.7)	1 (3.2)	12 (4.1)	
Condominium	1 (0.6)	0 (0.0)	1 (3.2)	2 (0.7)	
Other (e.g., townhouse, triplex)	4 (2.3)	1 (1.1)	4 (12.9)	9 (3.1)	
**Employment status**					0.001
Employed full-time	112 (65.5)	35 (39.8)	1 (3.2)	148 (51.0)	
Employed part-time	14 (4.1)	6 (6.8)	0 (0.0)	20 (6.9)	
Unemployed	7 (4.1)	6 (6.8)	0 (0.0)	13 (4.5)	
Retired or disabled	35 (20.5)	39 (44.3)	1 (3.2)	75 (25.9)	
**Household income range in 2017**					0.001
Less than $20,000	0 (0.0)	23 (26.1)	0 (0.0)	23 (7.9)	
$20,000 to $49,999	0 (0.0)	65 (73.9)	0 (0.0)	65 (22.4)	
$50,000 to $74,999	64 (37.4)	0 (0.0)	0 (0.0)	64 (22.1)	
Over $75,000	107 (62.6)	0 (0.0)	0 (0.0)	107 (36.9)	
**Discarded tires on property**					0.001
Yes	8 (4.7)	3 (3.4)	0 (0.0)	11 (3.8)	
No	152 (88.9)	78 (88.6)	19 (61.3)	249 (85.9)	
**Frequency of mosquito bites at home**					0.001
Very frequency	28 (16.4)	19 (21.6)	2 (6.5)	49 (16.9)	
Frequently	45 (26.3)	29 (33.0)	1 (3.2)	75 (25.9)	
Occasionally	79 (46.2)	32 (36.4)	6 (19.4)	117 (40.3)	
Seldon or never	17 (9.9)	7 (8.0)	0 (0.0)	24 (8.3)	
**Will report illegal dumping if saw one**					0.001
Very likely or likely	72 (42.1)	46 (52.3)	8 (25.8)	126 (43.4)	
Somewhat likely	95 (55.6)	42 (47.7)	12 (38.7)	149 (51.4)	
Note Likely					
**Willing to participant in tire clean-up**					0.001
Very willing or willing	97	56	16	169	
Somewhat willing or not willing	69 (40.4)	27 (30.7)	4 (12.9)	100 (34.5)	

### Illegally used vehicle tire sightings and preferred methods of communication

**Figure 1:**
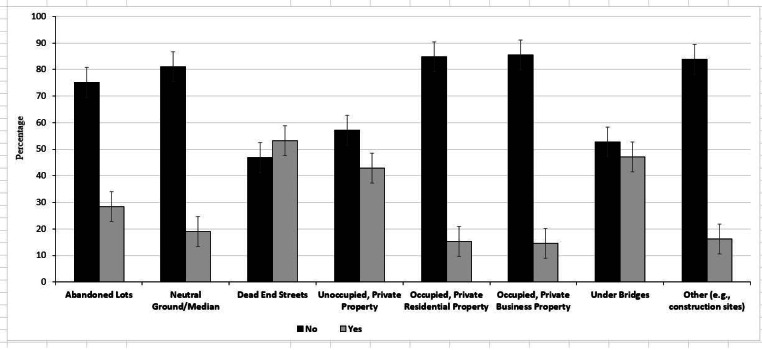
Distribution of New Orleans residents based on source of information regarding health information. Error bars represent 95% confidence intervals.

**Figure 2:**
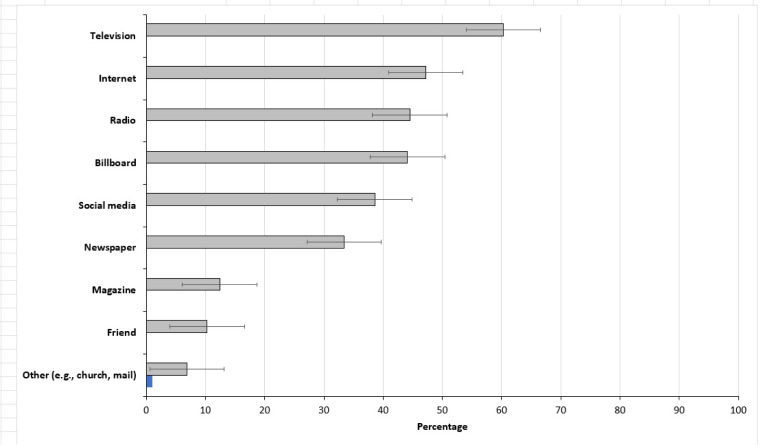
Preferred sources for receiving health information. Error bars represent 95% confidence intervals.

Over half (53.1%) of respondents had seen an illegally discarded vehicle tire (s) on dead end streets, under bridges (47.2%) and/or on unoccupied, private properties (42.8%) (Fig. 1). Figure 2 provides an overview of the preferred sources of public health information. Most respondents preferred to receive public health information via the television (60.3%), internet (47.2), radio (44.5%) or billboards (44.1%). None of the sources of information were statistically significant between the two household median income levels.

### Knowledge of mosquito-borne disease transmission and tire disposal

The differences between knowledge measures and median household income levels are highlighted in Table 2. Nearly eight in 10 respondents (78.3%) knew that mosquito-borne diseases can be spread through the bite of mosquitoes, and almost half of respondents did not know that mosquitoes can breed in polluted water. In addition, almost two out of three (64%) respondents indicated not knowing that the City of New Orleans will pick up to four tires placed next to garbage cans from properties eligible for garbage collection by the City, every second collection of the week for properties outside of the French Quarter/DDD and on Wednesdays for properties inside of the French Quarter/DDD. Only 2.9% of high median income households and 1.1% of low-income respondents achieved at least 80% on the knowledge score or answering at least one correct answer (good knowledge, *p* < 0.001).

**Table 2:** Knowledge of illegal tire discarding and associated mosquito disease risk among New Orleans, Louisiana residents.

**Variables**	**High-income residents** ***n* (%)**	**Low-income residents** ***n* (%)**	**Missing** ***n* (%)**	**Total** ***N* (%)**	***p*-value**
Mosquito disease can spread via mosquito bite					0.001
**Yes**	156 (91.2)	71 (80.7)	6 (19.4)	233 (80.3)	
No	15 (8.8)	17 (19.3)	25 (80.6)	57 (19.7)	
Mosquitoes can breed in polluted water					0.001
**Yes**	73 (42.7)	42 (47.7)	3 (9.7)	118 (40.7)	
No	98 (57.3)	46 (52.3)	28 (90.3)	172 (59.3)	
Mosquito disease can be transmitted via body fluids					0.464
**Yes**	39 (22.8)	19 (21.6)	4 (12.9)	62 (21.4)	
No	132 (77.2)	69 (78.4)	27 (87.1)	228 (78.6)	
Mosquito disease can be transmitted contaminated food					0.631
**Yes**	22 (12.9)	8 (9.1)	3 (9.7)	33 (11.4)	
No	149 (87.1)	80 (90.9)	28 (90.3)	257 (88.6)	
Other transmission routes					0.014
**Yes**	30 (17.5)	27 (30.7)	3 (9.7)	60 (20.7)	
No	∼	∼	∼	∼	
The city picks up tires (up to 4 tires) twice a month on pick up routes?					0.191
**Yes**	52 (30.4)	21 (23.9)	5 (16.1)	78 (26.9)	
No	119 (69.6)	67 (76.1)	26 (83.9)	212 (73.1)	
Overall, Knowledge level					0.001
Low knowledge	155 (90.6)	83 (94.3)	14 (45.2)	252 (86.9)	
**High Knowledge**	5 (2.9)	1 (1.1)	1 (3.2)	7 (2.4)	
Moderate knowledge	10 (5.8)	4 (0.3)	0 (0.0)	14 (4.8)	

**Notes.**

Note: All *P*-values are based on chi-square analysis of numbers by respondent income levels except those indicated by an asterisk (*) which are based on Fisher’s exact test. Bold fonts indicate correct answer. Overall knowledge score is based on the percentage of correct answers the overall score of all correct responses of the 12 questions that comprised this domain, with 80% or high indicating high knowledge.

### Attitude towards source reduction and mosquito control

Table 3 summarizes respondents’ attitudes regarding source reduction and mosquito control. Almost all (95.5%) respondents strongly agree or agree that mosquitoes are a serious disease. Thus, about 70.4% of respondents and 94.3% of respondents strongly agree or agree that spraying and mosquito control can prevent mosquito populations and disease. Most of the respondents (81.7%) also acknowledged responsibility to control mosquito breeding in their yards while about half, 51.1% of the respondents believe it is the city’s responsibility to control mosquitoes. Respondents’ overall attitude towards illegal tire discarding and source reduction differed significantly by median household income (*p* < 0.05); compared to low-income respondents, those with high-median household income 31.6% *vs.* 35.2% had negative attitude, 30.4% *vs.* 31.8% had positive attitude and 37.3% *vs.* 31.8% had moderate attitude.

**Table 3:** Attitudes towards source reduction and mosquito control among New Orleans, Louisiana residents (*n* = 290).

**Variables**	**High-income residents** ***n* (%)**	**Low-income residents** ***n* (%)**	**Missing** ***n* (%)**	**Total** ***N* (%)**	***p*-value**
Mosquito-borne diseases are a serious public health issue					0.001
**Strongly agree or agree**	164 (95.9)	84 (95.5)	5 (16.1)	253 (87.2)	
Strongly disagree or disagree	5 (2.9)	4 (1.4)	0 (0.0)	9 (3.1)	
Spraying controls mosquito populations					
**Strongly agree or agree**	120 (70.2)	61 (69.3)	3 (9.7)	184 (63.4)	0.001
Strongly disagree or disagree	45 (26.3)	26 (29.5)	2 (6.5)	73 (25.2)	
Control prevents mosquito-borne diseases					
**Strongly agree or agree**	164 (95.9)	83 (94.3)	4 (12.9)	251 (86.6)	0.000
Strongly disagree or disagree	6 (3.5)	4 (1.4)	1 (3.2)	11 (3.8)	
It is the city government’s responsible to control mosquitoes in my home or yard					0.001
**Strongly agree or agree**	91 (53.2)	44 (50.0)	2 (6.5)	137 (47.2)	
Strongly disagree or disagree	76 (44.4)	44 (50.0)	3 (9.7)	123 (42.4)	
It is my responsibility to control mosquito breeding in my yard					0.001
**Strongly agree or agree**	159 (93.0)	78 (88.6)	5 (16.1)	242 (83.4)	
Strongly disagree or disagree	12 (7.0)	10 (11.4)	26 (83.9)	48 (16.5)	
Overall, Attitude level					0.001
Negative attitude	54 (31.6)	31 (35.2)	11 (35.5)	96 (33.1)	
**Positive attitude**	52 (30.4)	28 (31.8)	1 (3.2)	81 (27.9)	
Moderate attitude	63 (37.3)	28 (31.8)	0 (0.0)	91 (31.4)	

**Notes.**

Note: All *P*-values are based on chi-square analysis of numbers by respondent income levels except those indicated by an asterisk (*) which are based on Fisher’s exact test. Bold fonts indicate correct answer. Overall attitude score is based on the percentage of correct answers the overall score of all correct responses of the seven questions that comprised this domain, with 80% or high indicating good or poor attitudes.

Surprisingly, when asked about how likely respondents are to report an illegal tire dumping, more than one in three respondents (34.0%) indicated not likely, and about 20.0% of respondents indicated somewhat likely to report an illegally used vehicle tire dumping. Similarly, 42% of the respondents indicated they were not willing to participate in a volunteer program to pick up used vehicle tires during neighborhood tire clean-up initiatives (Results not shown).

### Preventive measures against source reduction and mosquito bites

Table 4 shows that most respondents (81.0%) believe that using mosquito repellents is the best preventative measure for the prevention of mosquito bites. Eliminating standing water around the house (84.8%) was the commonly practiced measure for source reduction. Regarding preventative scores, 19.1% of the respondents achieved at least 80% on the preventative practice score (good preventative practice), and only 20.5% obtained at least 80% on the for preventative practice score for source reduction (good source reduction).

**Table 4:** Preventative measures against mosquito transmission and bites among New Orleans, Louisiana resident (*n* = 290).

**Variables**	**High-income residents** ***n* (%)**	**Low-income residents** ***n* (%)**	**Missing** ***n* (%)**	**Total** ***N* (%)**	***p*-value**
**Preventative mosquito bite measures**					
Use mosquito repellents					0.001
**Yes**	154 (90.1)	81 (92.0)	8 (25.8)	243 (83.8)	
No	17 (9.9)	7 (8.0)	23 (74.2)	47 (16.2)	
Use mosquito bed nets					0.008
**Yes**	43 (25.1)	11 (12.5)	2 (6.5)	56 (19.3)	
No	128 (74.9)	77 (87.5)	29 (93.5)	234 (80.7)	
Spraying by plane or track					
**Yes**					
No					
Use screens on windows and doors					0.001
**Yes**	128 (74.9)	62 (70.5)	7 (22.6)	197 (67.9)	
No	43 (25.1)	26 (29.5)	24 (77.4)	93 (32.1)	
Overall, preventative practices level					0.001
Poor practices	92 (53.8)	60 (68.2)	8 (25.8)	160 (55.2)	
**Good practices**	38 (22.2)	13 (14.8)	2 (6.5)	53 (18.3)	
Moderate practices	38 (22.2)	14 (15.9)	2 (6.5)	54 (18.6)	
**Source reduction measures**					
Eliminate standing water					0.001
**Yes**	161 (94.2)	85 (96.6)	8 (7.9)	254 (87.6)	
No	10 (5.8)	3 (3.4)	23 (74.2)	36 (12.4)	
Use of larvicide					0.004
**Yes**	80 (46.8)	28 (31.8)	6 (19.4)	114 (39.3)	
No	91 (53.2)	60 (68.2)	25 (80.6)	176 (60.7)	
Cleaning garbage and trash					0.261
**Yes**	39 (22.8)	24 (27.3)	4 (12.9)	67 (23.1)	
No	132 (77.2)	64 (72.7)	27 (87.1)	223 (76.9)	
Use mosquito eating fish					0.003
**Yes**	66 (38.6)	21 (23.9)	4 (12.9)	91 (31.4)	
No	105 (61.4)	67 (76.1)	27 (87.1)	199 (68.6)	
Spraying and fogging in yard					∼
**Yes**	89 (100)	37 (100)	8 (100)	134 (100)	
Other practices (e.g., bug lights, foliage)	3 (1.8)	5 (5.7)	1 (3.2)	9 (3.1)	
Overall, source reduction practices level					0.001
Poor practices	85 (49.7)	53 (60.2)	4 (12.9)	142 (49.0)	
**Good practices**	39 (22.8)	15 (17.0)	5 (16.1)	59 (20.3)	
Moderate practices	43 (25.1)	19 (21.6)	1 (3.2)	63 (21.7)	

**Notes.**

Note: All *P*-values are based on chi-square analysis of numbers by respondent income levels except those indicated by an asterisk (*) which are based on Fisher’s exact test. Bold fonts indicate correct answer. Overall preventative score is based on the percentage of correct answers the overall score of all correct responses of the five-source reduction and six mosquito bites questions that comprised this domain, with 80% or high indicating high good or poor. ∼ model did not converge.

### Predictors of knowledge and attitude levels (good *vs.* poor; positive *vs.* negative)

Table 5 presents the ordinal logistic regression analysis of the relationship between knowledge, attitudes, and respondents’ socio-economic variables. This table reveals significantly high knowledge regarding mosquito control and illegally discarded vehicle tires if a respondent was employed (OR: 1.00; 95% CI [0.000–0.006]), when all the other variables in the model are held constant. Similarly, we found increased odds of having a positive attitude if respondents owned their home (OR: 1.00; 95% CI [0.00–0.003]). All other variables are not significant.

**Table 5:** Predictors of knowledge and attitudes towards illegal tire discarding and source reduction among New Orleans Residents (*n* = 290).

	**Estimate**	**Std. Error**	**Wald**	**df**	**Sig.**	**OR (95% CI)**
**Knowledge**						
Low Knowledge	−2.116	9.477	0.050	1	0.823	0.12 (−20.69–16.458)
High Knowledge	−1.634	9.478	0.030	1	0.863	0.20 (−20.21–16.942)
Age	−0.004	0.009	0.154	1	0.694	1.00 (−0.022–0.014)
Sex	0.000	0.001	0.170	1	0.680	1.00 (−0.001–0.002)
Marital status	−0.013	0.017	0.546	1	0.460	0.99 (−0.046–0.021)
Employed	0.003	0.001	4.714	1	**0.030**	1.00 (0.000–0.006)
Educational level	0.001	0.002	0.215	1	0.643	1.00 (−0.002–0.004)
Home Ownership	−0.001	0.002	0.359	1	0.549	1.00 (−0.005–0.002)
Housing type	−0.001	0.001	1.465	1	0.226	1.00 (−0.002–0.000)
Low median household income	−4.326	9.429	0.210	1	0.646	0.01 (−22.807–14.156)
High median household income	−4.957	9.411	0.277	1	0.598	0.01 (−23.402–13.488)
**Attitude**						
Negative attitude	1.024	1.487	0.474	1	0.491	2.78 (−1.891–3.939)
Positive attitude	2.229	1.493	2.23	1	0.135	9.29 (−0.697–5.155)
Moderate attitude	4.753	1.520	9.778	1	**0.002**	115.93 (1.774–7.732)
Age	0.001	0.001	3.106	1	0.078	1.00 (0.00–0.003)
Sex	0.000	0.001	0.176	1	0.675	1.00 (−0.001–0.001)
Race	0.001	0.001	1.26	1	0.262	1.00 (−0.001–0.002)
Marital status	0.000	0.001	0.025	1	0.874	1.00 (−0.002–0.003)
Employed	0.000	0.001	0.022	1	0.882	1.00 (−0.002–0.002)
Educational level	0.001	0.001	0.817	1	0.366	1.00 (−0.002–0.002)
Home Ownership	0.002	0.001	5.925	1	**0.015**	1.00 (0.00–0.003)
Housing type	0.000	0.000	2.872	1	0.090	1.00 (−0.001–6.33E−05)
Low median household income	1.765	1.476	1.431	1	0.232	5.84 (−1.127–4.658)
High median household income	1.459	1.458	1.001	1	0.317	4.30 (−1.399–4.317)

**Notes.**

**Note**: Bold fonts indicate significant results.

OR Odds ratio CI Confidence intervals (*p* < 0.05)

## Discussion

While the City of New Orleans has made many efforts to combat illegal vehicle tire discarding and larviciding to control mosquito-borne and nuisance mosquitoes, illegal tire discarding continues to pose a threat to population and environmental health. Besides, *Ae. albopictus* was introduced to the US in contaminated tires after World War II, setting the stage for the transmission of arboviruses, that can cause both wildlife and human disease outbreaks such as dengue, chikungunya (Paupy et al., 2009) and La Crosse encephalitis (Gerhardt et al., 2001). Therefore, understanding and improving New Orleans residents’ knowledge, attitudes, and practices regarding illegally vehicle tire discarding, a potential habitat for mosquitoes and other pests, is essential to effectively prevent and control illegal tire discarding and mosquito borne-disease (Moise et al., 2013). An aim of this study was to assess the knowledge, attitudes, and practices of New Orleans residents regarding illegal vehicle tire discarding and their associated health risks. To the best of our knowledge, this is the first study to do so in continental US.

The current study found that there are relatively good attitudes and practices towards mosquito control among New Orleans residents despite a low level of knowledge regarding illegally discarded tires, a potential mosquito habitat. However, despite the low knowledge level overall, most respondents knew that mosquito disease can spread via mosquito bite (78.1%): although the proportion was high for respondents from high-income households (91.2%) compared to their counterparts in low-income households. The low knowledge finding in this study is contrary to that of Dowling et al. (2013) whose KAP study conducted in Washington, DC found greater knowledge among respondents with high socio-economic status but poor attitudes among respondents from middle and low-income socio-economic status. This result underscores the need for understanding site-specific mosquito risk, capacity of local stakeholders and community attitudes towards illegal tire discarding as a source of mosquitoes as there is no one-size-fits-all prevention approach to mosquito control, community education and empowerment (Morse et al., 2019). It also suggests a need for targeted and tailored mosquito interventions and ongoing awareness as necessary strategies to prevent used illegal tire discarding and source reduction to prevent exposure to mosquito-borne pathogens and bites.

Previous research has linked littering or illegal dumping to personal behaviors, environmental and systemic factors such as presence of more litter in an area (Arafat et al., 2007; Bateson et al., 2013), lack of waste management resources (Al-Khatib et al., 2015; Schultz et al., 2011), dumping convenience (Sokka, Antikainen & Kauppi, 2007), proximity to dump sites (Rupani et al., 2019), apathy and a lack of knowledge and awareness of local ordinances or poor enforcement (Khawaja & Shah, 2013; Zambezi, Muisa-Zikali & Utete, 2021). In addition, challenges pertaining to community perceptions of solid waste management have also been reported with mixed results. For example, one study found that while in some European countries, community involvement in the recycling and packaging cycle of waste helped curb the littering problem, in some countries, it contributed to the problem (Wolsink, 2010; Da Cruz, Simões & Marques, 2014). Other studies underscore the costs and benefits of recycling waste systems (Marques et al., 2014), regulation and reinforcement of waste facilities (*e.g.*, fees) (Mihai, 2012) as a hindrance.

We also found that two-thirds of respondents did not know that the City of New Orleans will pick up to 4 tires placed next to garbage cans from properties eligible for garbage collection by the City, every second collection of the week for properties outside of the French Quarter/DDD and on Wednesdays for properties inside of the French Quarter/DDD. Likewise, almost half of respondents (49.8%) said they are somewhat or unlikely to report an illegal tire dumping if they saw one and 35.7% were somewhat unwilling or unwilling to participate in a volunteer program to pick up tires in their neighborhood if the city organized such an event. This finding has important implications for developing used vehicle tire clean-up efforts because while such events can be conducted in New Orleans, finding volunteers will be a challenging task. It can therefore be assumed that raising awareness must be a high priority to heighten public knowledge to influence tire waste management positively and to mitigate potential health effects from illegal tire discarding.

This study also found that respondents from high-income households had favorable attitudes to mosquito activities (spaying, 70.2%, and control, 95.9%), source reduction practices and preventative measures against mosquito bites overall than respondents from low-income households. These results suggest that not being employed and having a low-income income can further limit home ownership, one’s capacity to practice preventative measures against mosquito bites or source reduction—factors that have also been linked to worse health outcomes (Beech et al., 2021; Kimmel et al., 2016). Previous studies have demonstrated how poverty can influence individual perceptions and behaviors (Wuepper & Lybbert, 2017). Here, we observed that being employed was associated with knowledge while home ownership was associated with attitudes towards illegal tire dumping. It may be that in low-income households, relative and absolute economic deficiency shape attitudes, and preventative practices in a manner that individuals focus on surviving rather than health. This also accords with several studies, which showed that people in poor neighborhoods are less healthy than their more affluent neighbors (Moise & Ruiz, 2016; Diez Roux, 2001; Diez Roux & Mair, 2010; Cohen et al., 2000).

There are limitations to this study that must be considered. First, this study was cross-sectional, and examined the KAP, mosquito control and illegal used vehicle tire relationships based on data collected at one point in time. In addition, because the study was based on subject self-selection, this may have contributed to non-respondents (24.5% of the sample did not respond to the survey). Therefore, with this data, it is impossible to fully eliminate sampling bias, fully understand KAP levels of those that did not respond to the survey as well as there is potential for underestimation in illegal discarded tire dumping sightings or KAP domain responses. Although a two-stage cluster random sampling procedure was employed to minimize selection bias, we surveyed only New Orleans households when tire discarding could have been carried out by residents of nearby parishes, which could have introduced selection bias. This is an important issue for future research. Further, we only included a single question in the survey in which different mosquito stages were listed, a common component of vector-borne disease KAP studies that would have provided a more thorough evaluation of a participant’s knowledge of mosquito biology and mosquito control. However, because the focus of this study was on examining the knowledge, attitudes, and practices towards mosquito control and illegally discarded waste vehicle tires, the current study adds to the value of previous KAP studies as well as tires and mosquito breeding (Kling, Juliano & Yee, 2007; Yee, Kneitel & Juliano, 2010; Eads, 1972; Lampman, Hanson & Novak, 1997).

In conclusion, this study established a low level of knowledge on illegal used tire dumping and mosquito control. Despite this low level of knowledge, the attitude and practice levels were modest. Despite home ownership, race and being employed, no other independent variables of interest were significant. Several questions remain unanswered at present. Compared to high income households, low-income households are at particularly high risk because they were found to have lower attitudes and practice levels towards illegal tire dumping and mosquito control. Therefore, there is an urgent need for massive place-based awareness campaigns and community engagement efforts to raise the knowledge of residents in New Orleans. The local mosquito control board should coordinate with the local public health department to develop targeted health promotion interventions regarding mosquito control and tires as well as design community level efforts to educate, inform, and mobilize communities to address the potential consequences of used tire dumping. Further work will assess these findings to examine effective communication strategies about these issues.

##  Supplemental Information

10.7717/peerj.14188/supp-1 Supplemental Information 1 Survey

10.7717/peerj.14188/supp-2 Data S1 Raw Data

## Abbreviations

ITNs: insecticide treated bed nets
US: United States
CI: Confidence Interval

## Additional Information and Declarations

### Data Availability

The following information was supplied regarding data availability: The raw data is available in the Supplemental Files.

## References

[ref-1] Al-Khatib IA, Kontogianni S, Abu Nabaa H, Alshami N, Al-Sari MI (2015). Public perception of hazardousness caused by current trends of municipal solid waste management. Waste Management.

[ref-2] Aliabadi BW, Juliano SA (2002). Escape from gregarine parasites affects the competitive interactions of an invasive mosquito. Biological Invasions.

[ref-3] Arafat HA, Al-Khatib IA, Daoud R, Shwahneh H (2007). Influence of socio-economic factors on street litter generation in the middle east: effects of education level, age, and type of residence. Waste Management & Research.

[ref-4] Aziz AT, Dieng H, Ahmad AH, Mahyoub JA, Turkistani AM, Mesed H, Koshike S, Satho T, Salmah MC, Ahmad H, Zuharah WF, Ramli AS, Miake F (2012). Household survey of container-breeding mosquitoes and climatic factors influencing the prevalence of aedes aegypti (diptera: Culicidae) in Makkah City, Saudi Arabia. Asian Pacific Journal of Tropical Biomedicine.

[ref-5] Bateson M, Callow L, Holmes JR, Redmond Roche ML, Nettle D (2013). Do images of ‘watching eyes’ induce behaviour that is more pro-social or more normative? A field experiment on littering. PLOS ONE.

[ref-6] Baumgartner DL (1988). Suburban accumulations of discarded tires in northeastern Illinois and their associated mosquitoes. Journal of the American Mosquito Control Association.

[ref-7] Beech BM, Ford C, Thorpe RJ, Bruce MA, Norris KC (2021). Poverty, racism, and the public health crisis in America. Frontiers in Public Health.

[ref-8] Beier JC, Patricoski C, Travis M, Kranzfelder J (1983). Influence of water chemical and environmental parameters on larval mosquito dynamics in tires. Environmental Entomology.

[ref-9] Bodner D, LaDeau SL, Biehler D, Kirchoff N, Leisnham PT (2016). Effectiveness of print education at reducing urban mosquito infestation through improved resident-based management. PLOS ONE.

[ref-10] Chen CC, Yamada T, Chiu IM, Liu YK (2009). Evaluation of the waste tire resources recovery program and environmental health policy in Taiwan. International Journal of Environmental Research and Public Health.

[ref-11] City of New Orleans (2019). Tire shop regulations. https://www.nola.gov/safety-and-permits/tire-shop-regulations/.

[ref-12] Cherry CC, Beer KD, Fulton C, Wong D, Buttke D, Staples JE, Ellis EM (2016). Knowledge and use of prevention measures for chikungunya virus among visitors—Virgin Islands National Park, 2015. Travel Medicine and Infectious Disease.

[ref-13] Cohen D, Spear S, Scribner R, Kissinger P, Mason K, Wildgen J (2000). Broken windows And the risk of gonorrhea. American Journal of Public Health.

[ref-14] Congressional Research Service (2008). Hurricane Katrina, continuing debris removal and disposal issues: status and associated issues.

[ref-15] Corrin T, Waddell L, Greig J, Young I, Hierlihy C, Mascarenhas M (2017). Risk perceptions, attitudes, and knowledge of Chikungunya among the public and health professionals: a systematic review. Tropical Medicine and Health.

[ref-16] Costanzo KS, Mormann K, Juliano SA (2005). Asymmetrical competition and patterns of abundance of *Aedes albopictus* and *Culex pipiens* (diptera: Culicidae). Journal of Medical Entomology.

[ref-17] Da Cruz NF, Simões P, Marques RC (2014). Costs and benefits of packaging waste recycling systems. Resources, Conservation and Recycling.

[ref-18] Davis R, Cook D, Cohen L (2005). A community resilience approach to reducing ethnic and racial disparities in health. American Journal of Public Health.

[ref-19] Diez Roux AV (2001). Investigating neighborhood and area effects on health. American Journal of Public Health.

[ref-20] Diez Roux AV, Mair C (2010). Neighborhoods and health. Annals of the New York Academy of Sciences.

[ref-21] Dowling Z, Armbruster P, LaDeau SL, De Cotiis M, Mottley J, Leisnham PT (2013). Linking mosquito infestation to resident socioeconomic status, knowledge, and source reduction practices in suburban Washington, DC. Ecohealth.

[ref-22] Eads R (1972). Recovery of *Aedes albopictus* from used tires shipped to United States ports. Mosquito News.

[ref-23] Elson WH, Ortega E, Kreutzberg-Martinez M, Jacquerioz F, Cabrera LN, Oberhelman RA, Paz-Soldan VA (2020). Cross-sectional study of dengue-related knowledge, attitudes and practices in Villa El Salvador, Lima, Peru. BMJ Open.

[ref-24] Farquhar SA, Michael YL, Wiggins N (2005). Building on leadership and social capital to create change in 2 urban communities. American Journal of Public Health.

[ref-25] Fernandes JN, Moise IK, Maranto GL, Beier JC (2018). Revamping mosquito-borne disease control to tackle future threats. Trends in Parasitology.

[ref-26] Flatie BT, Munshea A (2021). Knowledge, attitude, and practice towards malaria among people attending Mekaneeyesus Primary Hospital, South Gondar, northwestern Ethiopia: a cross-sectional study. Journal of Parasitology Research.

[ref-27] Gerhardt RR, Gottfried KL, Apperson CS, Davis BS, Erwin PC, Smith AB, Panella NA, Powell EE, Nasci RS (2001). First isolation of la crosse virus from naturally infected *Aedes albopictus*. Emerging Infectious Diseases.

[ref-28] González MA, Rodríguez-Sosa MA, Vásquez-Bautista YE, Rosario EDC, Durán-Tiburcio JC, Alarcón-Elbal PM (2020). A survey of tire-breeding mosquitoes (diptera: Culicidae) in the Dominican Republic: considerations about a pressing issue. Biomedica.

[ref-29] Haramis LD (1984). *Aedes triseriatus*: a comparison of density in tree holes vs. discarded tires. Mosquito News.

[ref-30] Hawley WA, Reiter P, Copeland RS, Pumpuni CB, Craig GB (1987). *Aedes albopictus* in North America: probable introduction in used tires from Northern Asia. Science.

[ref-31] Kala Chouakeu NA, Ngingahi LG, Bamou R, Talipouo A, Ngadjeu CS, Mayi MPA, Kopya E, Awono-Ambene P, Tchuinkam T, Antonio Nkondjio C (2021). Knowledge, attitude, and practices (kap) of human populations towards malaria control in four ecoepidemiological settings in cameroon. Japanese Journal of Tropical Medicine and Hygiene.

[ref-32] Khawaja FS, Shah A (2013). Determinants of littering: an experimental analysis. The Pakistan Development Review.

[ref-33] Kimmel PL, Fwu CW, Abbott KC, Ratner J, Eggers PW (2016). Racial disparities in poverty account for mortality differences in us medicare beneficiaries. SSM—Population Health.

[ref-34] Kling LJ, Juliano SA, Yee DA (2007). Larval mosquito communities in discarded vehicle tires in a forested and unforested site: Detritus type, amount, and water nutrient differences. Journal of Vector Ecology: Journal of the Society for Vector Ecology.

[ref-35] Lampman R, Hanson S, Novak R (1997). Seasonal abundance and distribution of mosquitoes at a rural waste tire site in Illinois. Journal of the American Mosquito Control Association.

[ref-36] Lewis JA, Zipperer WC, Ernstson H, Bernik B, Hazen R, Elmqvist T, Blum MJ (2017). Socioecological disparities in New Orleans following Hurricane Katrina. Ecosphere.

[ref-37] Manga IA, Gaye A, Dia A, Kouevidjin E, Dos Reis MR, Niang AS, Fall AN, Anquetil CM, Ndiaye JLA (2021). Knowledge, attitudes and practices (kap) to assess the impact of school children’s awareness of malaria using the Moski Kit^®^ tool: study case of some Dakar schools in Senegal. Malaria Journal.

[ref-38] Marques RC, Da Cruz NF, Simões P, Faria Ferreira S, Pereira MC, De Jaeger S (2014). Economic viability of packaging waste recycling systems: a comparison between Belgium and Portugal. Resources, Conservation and Recycling.

[ref-39] Mihai FC (2012). Population access to waste collection services: urban vs rural areas in Romania. Bulletin of University of Agricultural Sciences and Veterinary Medicine Cluj-Napoca Agriculture.

[ref-40] Moise IK, Brown KS, Riegel C, Kalipeni E, Ruiz V (2013). Geographic assessment of unattended swimming pools in post-Katrina New Orleans, 2006-2008. Annals of the Association of American Geographers.

[ref-41] Moise IK, Kangmennaang J, Hutchings TCSG, Sheskin IM, Fuller DO (2018). Perceptions of Zika virus risk during 2016 outbreak, Miami-Dade county, Florida, USA. Emerging Infectious Diseases.

[ref-42] Moise IK, Ortiz-Whittingham LR, Omachonu V, Clark M, Xue RD (2021). Fighting mosquito bite during a crisis: capabilities of Florida mosquito control districts during the COVID-19 pandemic. BMC Public Health.

[ref-43] Moise IK, Riegel C, Muturi EJ (2018). Environmental and social-demographic predictors of the southern house mosquito culex quinquefasciatus in new orleans, louisiana. Parasites & Vectors.

[ref-44] Moise IK, Ruiz MO (2016). Hospitalizations for substance abuse disorders before and after Hurricane Katrina: spatial clustering and area-level predictors, New Orleans, 2004 and 2008. Preventing Chronic Disease.

[ref-45] Moise I, Sheskin I, Fuller D (submitted). Zika-related knowledge, and attitudes, and practices among Miami-Dade residents during the 2016 outbreak of the Zika virus. EID.

[ref-46] Morse W, Izenour K, McKenzie B, Lessard S, Zohdy S (2019). Perceptions and practices of mosquito-borne diseases in Alabama—is concern where it should be?. BMC Public Health.

[ref-47] New Orleans City Planning Commission (2016). The context: previous planning and the charter amendment. New Orleans’ 2030 Plan. https://masterplan.nola.gov/volume-2/3/.

[ref-48] Paupy C, Delatte H, Beilhe LBagny, Corbel V, Fontenille D (2009). *Aedes albopictus*, an arbovirus vector: from the darkness to the light. Microbes and Infection.

[ref-49] Peyton E, Campbell S, Candeletti T, Romanowski M, Crans W (1999). Aedes (finlaya) japonicus japonicus (theobald), a new introduction into the United States. Journal of the American Mosquito Control Association.

[ref-50] Phuyal P, Kramer IM, Kuch U, Magdeburg A, Groneberg DA, Lamichhane Dhimal M, Montag D, Harapan H, Wouters E, Jha AK, Dhimal M, Müller R (2022). The knowledge, attitude and practice of community people on Dengue Fever in Central Nepal: a cross-sectional study. BMC Infectious Diseases.

[ref-51] Pogreba-Brown K, Austhof E, Okello A, Weiss J, Lira R, Ernst K (2019). Public perceptions of non-pharmaceutical interventions for influenza and mosquito-borne illnesses—a statewide survey in Arizona. Perspectives in Public Health.

[ref-52] Regmi K, Kunwar A, Ortega L (2016). A systematic review of knowledge, attitudes and beliefs about malaria among the South Asian population. Infection Ecology & Epidemiology.

[ref-53] Remoundou K, Koundouri P (2009). Environmental effects on public health: An economic perspective. International Journal of Environmental Research and Public Health.

[ref-54] Rupani PF, Maleki Delarestaghi R, Asadi H, Rezania S, Park J, Abbaspour M, Shao W (2019). Current scenario of the Tehran municipal solid waste handling rules towards green technology. International Journal of Environmental Research and Public Health.

[ref-55] Sallam MF, Michaels SR, Riegel C, Pereira RM, Zipperer W, Lockaby BG, Koehler PG (2017). Spatio-temporal distribution of vector-host contact (vhc) ratios and ecological niche modeling of the West Nile Virus mosquito vector, *Culex quinquefasciatus*, in the City of New Orleans, LA, USA. International Journal of Environmental Research and Public Health.

[ref-56] Smart Methodology (2012). Sampling for smart including considerations for urban sampling. https://smartmethodology.org/survey-planning-tools/smart-methodology/smart-methodology-paper.

[ref-57] Schultz PW, Bator RJ, Large LB, Bruni CM, Tabanico JJ (2011). Littering in context: personal and environmental predictors of littering behavior. Environment and Behavior.

[ref-58] Selvarajoo S, Liew JWK, Tan W, Lim XY, Refai WF, Zaki RA, Sethi N, Wan Sulaiman WY, Lim YAL, Vadivelu J, Vythilingam I (2020). Knowledge, attitude and practice on Dengue prevention and Dengue seroprevalence in a Dengue hotspot in Malaysia: a cross-sectional study. Scientific Reports.

[ref-59] Sokka L, Antikainen R, Kauppi PE (2007). Municipal solid waste production and composition in Finland—changes in the period 1960–2002 and prospects until 2020. Resources, Conservation and Recycling.

[ref-60] State of Louisiana (2011). Louisiana laws revised statutes title 30—minerals, oil, and gas and environmental quality rs 30:2418—waste tires. LA Rev Stat §30:2418. https://law.justia.com/codes/louisiana/2020/revised-statutes/title-30/rs-2418/.

[ref-61] Tuiten W, Koenraadt CJM, McComas K, Harrington LC (2009). The effect of west nile virus perceptions and knowledge on protective behavior and mosquito breeding in residential yards in upstate New York. EcoHealth.

[ref-62] Walker KR, Williamson D, Carrière Y, Reyes-Castro PA, Haenchen S, Hayden MH, Jeffrey Gutierrez E, Ernst KC (2018). Socioeconomic and human behavioral factors associated with *Aedes aegypti* (diptera: Culicidae) immature habitat in Tucson, AZ. Journal of Medical Entomology.

[ref-63] WDSU (2021). New Orleans east roadway continues to be plagued by illegal dumping. https://www.wdsu.com/article/new-orleans-east-illegal-dump-site-investigation/38475060.

[ref-64] Weave MP (2006). Fear and loathing in Post-Katrina emergency debris management: according to whom, pursuant to what, and you want to dump that where comment. Tulane Environmental Law Journal.

[ref-65] WGNO (2020). Property owner upset that 200 tires were illegally dumped on her property. https://wgno.com/news/property-owner-upset-that-200-tires-were-illegally-dumped-on-her-property/.

[ref-66] Wolsink M (2010). Contested environmental policy infrastructure: socio-political acceptance of renewable energy, water, and waste facilities. Environmental Impact Assessment Review.

[ref-67] Wuepper D, Lybbert TJ (2017). Perceived self-efficacy, poverty, and economic development. Annual Review of Resource Economics.

[ref-68] Yee D (2008). Tires as habitats for mosquitoes: a review of studies within the eastern United States. Journal of Medical Entomology.

[ref-69] Yee DA, Kneitel JM, Juliano SA (2010). Environmental correlates of abundances of mosquito species and stages in discarded vehicle tires. Journal of Medical Entomology.

[ref-70] Zambezi FM, Muisa-Zikali N, Utete B (2021). Effectiveness of community participation as anti-litter monitors in solid waste management in metropolitan areas in a developing country. Environment, Development and Sustainability.

