# Supplementary material for: Knowledge, attitudes, and practices towards mosquito control and used vehicle tire dumping by median household income, in metropolitan New Orleans, Louisiana
Source: PeerJ. 2022 Dec 2;10:e14188. doi: 10.7717/peerj.14188 (PMC9744171; doi:10.7717/peerj.14188)
Supplement: Supplemental Information 1 [file peerj-10-14188-s001.pdf]

CITY OF NEW ORLEANS
MOSQUITO, TERMITE AND RODENT CONTROL
BOARD & SANITATION DEPARTMENT

MIAMI

New Orleans Residents' Perspectives on Illegal Tire Dumping

Thank you for agreeing to participate in this study, which aims to understand the extent of used tire dumping in the City of New Orleans. Your feedback will help the city to identify and target areas for mosquito control efforts.

Do you store/have used or discarded tires on your property?

○ Yes ○ No

Where do you most often see disposed used tires?
Please select all that apply.

- ☐ Abandoned lots
- ☐ On the neutral ground/median
- ☐ Dead end streets
- ☐ Unoccupied, private property
- ☐ Occupied, private residential property
- ☐ Occupied, private business property
- ☐ Under bridges
- ☐ Other: ____________

How likely are you to report an illegal used tire dumping if you saw one?

○ Very likely ○ Likely ○ Somewhat likely ○ Not likely

How willing would you be to participate in a volunteer program to pick up used tires in your neighborhood?

○ Very willing ○ Willing ○ Somewhat willing ○ Not willing

Do you know that the City will pick up up to 4 tires placed next to garbage cans from properties eligible for garbage collection by the City, every second collection of the week for properties outside of the French Quarter/DDD and on Wednesdays for properties inside of the French Quarter/DDD?

○ Yes ○ No

These next questions ask about your perceptions related to the type of communication that should be used by the City of New Orleans to discourage people from illegal used tire dumping and how you would prefer to receive such messages.

How effective are the following strategies at reducing the number of used tires dumped illegally?

	Very Ineffective	Ineffective	Effective	Very Effective
Publicize that used or waste tires damages the image of our community	○	○	○	○
Publicize that used tires can be a significant breeding source for disease carrying mosquitoes if improperly stored and allowed to accumulate water	○	○	○	○
Tell citizens that they can report illegal dumping by calling 311	○	○	○	○
Use a famous person from the area to be an anti-used tire dumping spokesperson	○	○	○	○
Tell people that dumping used tires is not the right thing to do	○	○	○	○
Remind people that dumping is illegal and violators are subject to fines and criminal prosecution	○	○	○	○
Have a slogan that evokes community pride	○	○	○	○

How would you like to receive information about public health and tire dumping?
Please select all that apply.

- ☐ Social Media
- ☐ Radio
- ☐ Television
- ☐ Newspaper
- ☐ Internet
- ☐ Magazine
- ☐ Billboard
- ☐ Friend
- ☐ Other ________

These next questions ask about your perceptions and knowledge related to mosquito-borne diseases.

What is the mechanism of transmission of mosquito-borne diseases?
Please select all that apply.

Body fluids	Polluted water	Contaminated food	Mosquito bite	Other
☐	☐	☐	☐	☐

How often do mosquitoes bite you around your home?
Please select all that apply.

Very frequently	Frequently	Occasionally	Seldom or Never
○	○	○	○

Which are mosquito-breeding sites?
Please select all that apply.

Tires	Hollow trees	Gutters	In walls	On garbage	Other
☐	☐	☐	☐	☐	☐

Which of these preventive measures do you consider more effective to avoid mosquito eggs from hatching of the reproduction of larvae and pupae?
Please select all that apply.

- ☐ Eliminate standing water
- ☐ Use of larvicide
- ☐ Wash water containers
- ☐ Use of fish in water containers
- ☐ Spraying/fogging your yard
- ☐ Other ______

Which of these measures do you consider more effective to prevent mosquito bites?
Please select all that apply.

- ☐ Use of insect repellent
- ☐ Use of bed nets
- ☐ Use of insecticides by plane or truck
- ☐ Cover water containers
- ☐ Use of screens in window and doors
- ☐ Backyard spray treatment

These next questions ask about your perceptions related to preventative measures to prevent the risks of mosquito-borne diseases.

	Strongly agree	Agree	Disagree	Strongly disagree
Mosquito borne diseases are a serious health problem.	○	○	○	○
Spraying is the best method to reduce mosquito populations.	○	○	○	○
Controlling mosquito populations can reduce mosquito-borne diseases.	○	○	○	○
The City of New Orleans is responsible for reducing the number of mosquito larvae in resident's homes.	○	○	○	○
It is my responsibility to prevent the spread of mosquito larvae in my home	○	○	○	○

Preventive Measures Against MBDs

These questions ask about the preventative measures that you take to prevent yourself and your family against mosquito-borne diseases.

Which practices for preventing mosquito breeding are you using?
Please select all that apply.

- ☐ Eliminate standing water
- ☐ Use of larvicide
- ☐ Wash water containers
- ☐ Use of fish in water containers
- ☐ Spraying/fogging your yard
- ☐ Other ________

Which practices for preventing mosquito bites are you using?
Please select all that apply.

- ☐ Use of insecticide
- ☐ Use of repellent
- ☐ Use of screens in window and doors
- ☐ Use of bed nets
- ☐ Other ________

Lastly, we'd like to ask you a few questions about your background.

What is your age?

18 to 24	25 to 34	35 to 44	45 to 54	Over 55
◯	◯	◯	◯	◯

What is the highest level of education you have completed?

- ◯ Less than a high school diploma
- ◯ Some college
- ◯ Bachelor's degree (e.g. BA, BS)
- ◯ Graduate degree (e.g. MA, Msc, PhD, JD, MD)

What is your 5-digit home zip code?

[ ]

To which sex do you most identify?

Male	Female	Other
◯	◯	◯

What is your marital status?

- ◯ Single (never married)
- ◯ Married or in a domestic partnership
- ◯ Widowed, divorced, or separated
- ◯ Other [ ]

What is your current employment status?

- ◯ Employed full time
- ◯ Employed part time
- ◯ Unemployed
- ◯ Retired or disabled

What was your household income range (before taxes) in 2017?

Less than $20,000	$20,000 to $49,999	$50,000 to $74,999	Over $75,000
◯	◯	◯	◯

How do you identify yourself? Please select all that apply.

- ☐ Vietnamese
- ☐ Middle Eastern
- ☐ Hispanic/Latinx
- ☐ American Indian or Alaska Native
- ☐ Asian
- ☐ Black or African American
- ☐ Native Hawaiian or Other Pacific Islander
- ☐ White
- ☐ Two or more races
- ☐ Other ________

How would you describe the home you currently live in?

- ○ Single family home
- ○ Duplex
- ○ Apartment
- ○ Condominium
- ○ Mobile home
- ○ Other ________

How would you describe the home you currently live in?

Owner occupied	Rental	Other
○	○	○

Would you like to be included in our email database to receive updates on the upcoming anti-used or waste tire program in New Orleans?

Yes	No
○	○

What is your email address?

____
